# Use of Thermal Ablation in Low-Resource Settings: Experience From Three Multicenter Noninferiority Randomized Clinical Trials

**DOI:** 10.1200/GO-25-00050

**Published:** 2025-06-06

**Authors:** Gabriel Conzuelo Rodriguez, Miriam L. Cremer, Richard Muwonge, Partha Basu

**Affiliations:** ^1^ Basic Health International, Pittsburgh, PA; ^2^ Cleveland Clinic Lerner College of Medicine, Cleveland, OH; ^3^ International Agency for Research on Cancer, Lyon, France

## Abstract

**PURPOSE:**

Thermal ablation (TA) is now a widely used treatment for cervical precancer in low- and middle-income countries. Over the past decade, TA devices have been redesigned to be more portable, user-friendly, and affordable. This analysis combines data from three large randomized clinical trials comparing the efficacy, safety, and acceptability of TA with those of the previous standard, gas-based cryotherapy.

**METHODS:**

This analysis used Human Papillomavirus (HPV) test results at 1-year post-treatment as the primary outcome. Secondary outcomes included side effects and patient satisfaction.

**RESULTS:**

Of the 2,948 participants treated with either TA or gas-based cryotherapy, 80.9% and 81.8% completed HPV testing at 1 year, respectively. Overall, 60.7% tested negative for HPV at follow-up, with slightly higher rates in the TA group (62.5%) compared with cryotherapy (58.7%), although the difference was not statistically significant (*P* value = .14). Side effects were minimal for both treatments. Severe pain was slightly more common with TA (7.6% *v* 3.9% for cryotherapy), but vasovagal responses were less frequent (2.3% *v* 7.6%). Satisfaction with treatment was high (approximately 98%) across both groups.

**CONCLUSION:**

Our findings support the efficacy of TA in treating cervical precancer, offering an effective and practical alternative in low-resource settings. However, future research is urgently needed to address critical questions, including the standardization of treatment protocols and tailored approaches for women living with HIV.

## INTRODUCTION

Cervical cancer is a leading cause of cancer-related death in low- and middle-income countries (LMICs), accounting for approximately 90% of mortality.^1^ Unlike other malignant neoplasias, cervical cancer is preceded by the development of precancerous lesions, known as cervical intraepithelial neoplasia grade 2 or higher (CIN2+). These lesions take years to progress into invasive cancer and can be effectively treated using ablation techniques.^2^ Timely treatment with ablation could help lower cervical cancer mortality in LMICs.

**CONTEXT**

**Key Objective**
To describe the experience of using thermal ablation (TA) in low-resource settings across four countries: China, Colombia, El Salvador, and Zambia.
**Knowledge Generated**
Across different settings, the proportion of women with a negative human papillomavirus (HPV) test during follow-up visits was comparable between TA and standard gas-based cryotherapy (62.5% *v* 58.7%), supporting the efficacy of this treatment modality. Furthermore, TA was well tolerated, as indicated by the levels of pain experienced during treatment and the low incidence of side effects. Finally, most women who underwent TA were very satisfied and would recommend it to others.
**Relevance**
TA is a viable alternative to treat cervical precancer. Future studies should seek to standardize the treatment protocol and assess its effectiveness among women living with HIV.

For many years, gas-based cryotherapy was the standard-of-care precancer treatment in LMICs. However, the prohibitive costs of medical-grade gas, along with challenges in procuring and transporting gas tanks, have hindered widespread use of cryotherapy in many settings.^3,4^ As a result, its contribution in scaling up cervical screening programs in the LMICs was minimal. Over the past decade, an older ablation technique known as thermal ablation (TA) was redesigned to address some of the challenges associated with cryotherapy.^5^ New devices were designed to be lightweight, compact, and battery-operated, which significantly enhances their portability.^5^ In addition, TA eliminates the need for gas or other consumables, making it a more practical and sustainable option.

The Affordable Cancer Technologies (ACTs) program of the Center for Global Health of the National Cancer Institute (USA) supports innovative research on issues relevant to global cancer control. Within the framework of ACT program, International Agency for Research on Cancer collaborated with LIGER INC (Lehi, Utah) to develop a compact battery-driven TA from a prototype stage and evaluated its safety and efficacy in a randomized trial nested in the screen-and-treat program in Zambia (ClinicalTrials.gov identifier: NCT02956239).^6^ Similarly, Basic Health International, through subcontracts with Cleveland Clinic, collaborated with WiSAP (Brunnthal, Germany) to redesign their nonportable device used since the 1970s. Two trials were developed to assess the efficacy of two designs of a portable battery-operated device for biopsy-confirmed treatment of CIN2/3 (ClinicalTrials.gov identifiers: NCT03084081, NCT02956239). The protocols of the two studies, NCT03084081 and NCT03429582, were evaluated and approved by the Cleveland Clinic Institutional Review Board. The protocol of the third study, 1UH2CA202721-01, was reviewed and approved by the research ethics committees at IARC and the University of North Carolina at Chappel Hill. In addition, all studies received approval from local ethics committees at participating sites in China, Colombia, El Salvador, and Zambia. All participants provided written consent, with those unable to read or write providing a fingerprint with a delegate witness present.

The objective of this article is to describe the experience of using TA in low-resource settings across four countries: China, Colombia, El Salvador, and Zambia. We provided estimates of the efficacy based on human papillomavirus (HPV) testing at 1-year post-treatment and safety and acceptability of this procedure compared with those of the previous standard, gas-based cryotherapy. Finally, we explored the future of this technology, highlighting key questions to address and challenges to overcome.

## METHODS

### Study Sample

This study incorporates data from three noninferiority randomized clinical trials, each comparing the efficacy, safety, and acceptability of TA against those of gas-based cryotherapy and/or excisional treatment. Together, these studies enrolled a total of 5,675 women across four countries: China (n = 1,461), Colombia (n = 106), El Salvador (n = 984), and Zambia (n = 3,124). Although the inclusion and exclusion criteria of the studies were similar, an important distinction was that enrollment required a CIN2+ diagnosis in China, Colombia, and El Salvador, whereas in Zambia, only a positive visual inspection with acid (VIA) test was necessary. Nevertheless, all studies performed the VIA test and collected samples for HPV testing during the baseline visit. Another important difference between studies was that only participants from Zambia were tested for HIV status. A summary of the studies is presented in Table 1.

**TABLE 1:** Summary of the Three Studies

Characteristic	Study Identification Number		
	NCT03084081	NCT03429582 ^a^	NCT02956239
Site(s)	El Salvador, Colombia, China	El Salvador, China	Zambia
Enrollment	1,132 (CO_2_ = 377; TA = 378)	1,154 (CO_2_ = 386; TA single tip = 378; TA multiple tips = 384)	1,161 (NO_2_ = 533; TA = 506)
Inclusion criteria	Age 20-59 years CIN2+	Age 20-59 years CIN2+	Age 25-59 years VIA+ HPV+
Exclusion criteria	Pregnancy Previous surgery of the cervix Not able to provide informed consent	Pregnancy Previous surgery of the cervix Not able to provide informed consent	Pregnancy Not able to provide informed consent
Gas-based cryotherapy protocol (gas)	Double-freeze (CO_2_)	Double-freeze (CO_2_)	Double-freeze (NO_2_)
TA protocol^b^	Single tip: 19-mm conical (40 seconds)	Single tip: 16-mm conical (20 seconds) Multiple tips: 10-mm shallow endocervical (20 seconds), then 16-mm flat ectocervical (20 seconds) overlapping fields	Single tip: 19-mm flat (30 seconds)
TA manufacturer	WiSAP	WiSAP	Liger medical

NOTE. Gas-based cryotherapy with CO_2_ or NO_2_.

Abbreviations: CIN2+, cervical intraepithelial neoplasia grade 2 or higher; CO_2_, carbon dioxide; HPV, human papillomavirus; NO_2_, nitrous oxide; TA, thermal ablation; VIA, visual inspection with acid.

^a^ Ongoing study.

^b^All protocols allowed for extra 20 seconds applications to cover the entire transformation zone.

**FIG A1:**
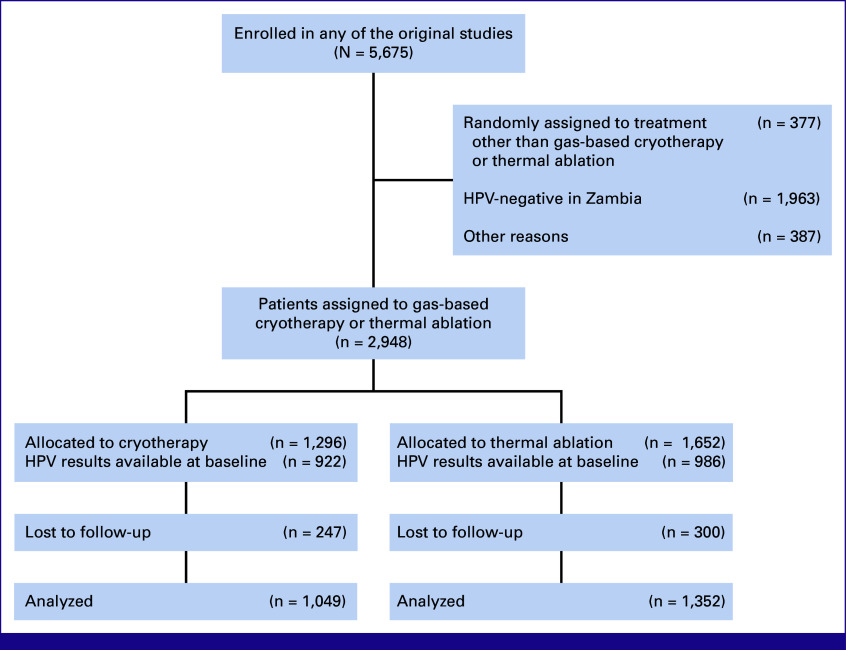
CONSORT diagram. HPV, human papillomavirus.

The present analysis was restricted to women who were randomly assigned to either gas-based cryotherapy or TA. In addition, and only for the Zambia site, we further restricted our analysis to women with a positive HPV test at baseline. Patients from China, Colombia, and El Salvador had CIN2+ and are presumed to have HPV. See Appendix Figure A1 for details.

### Procedures

**FIG 1:**
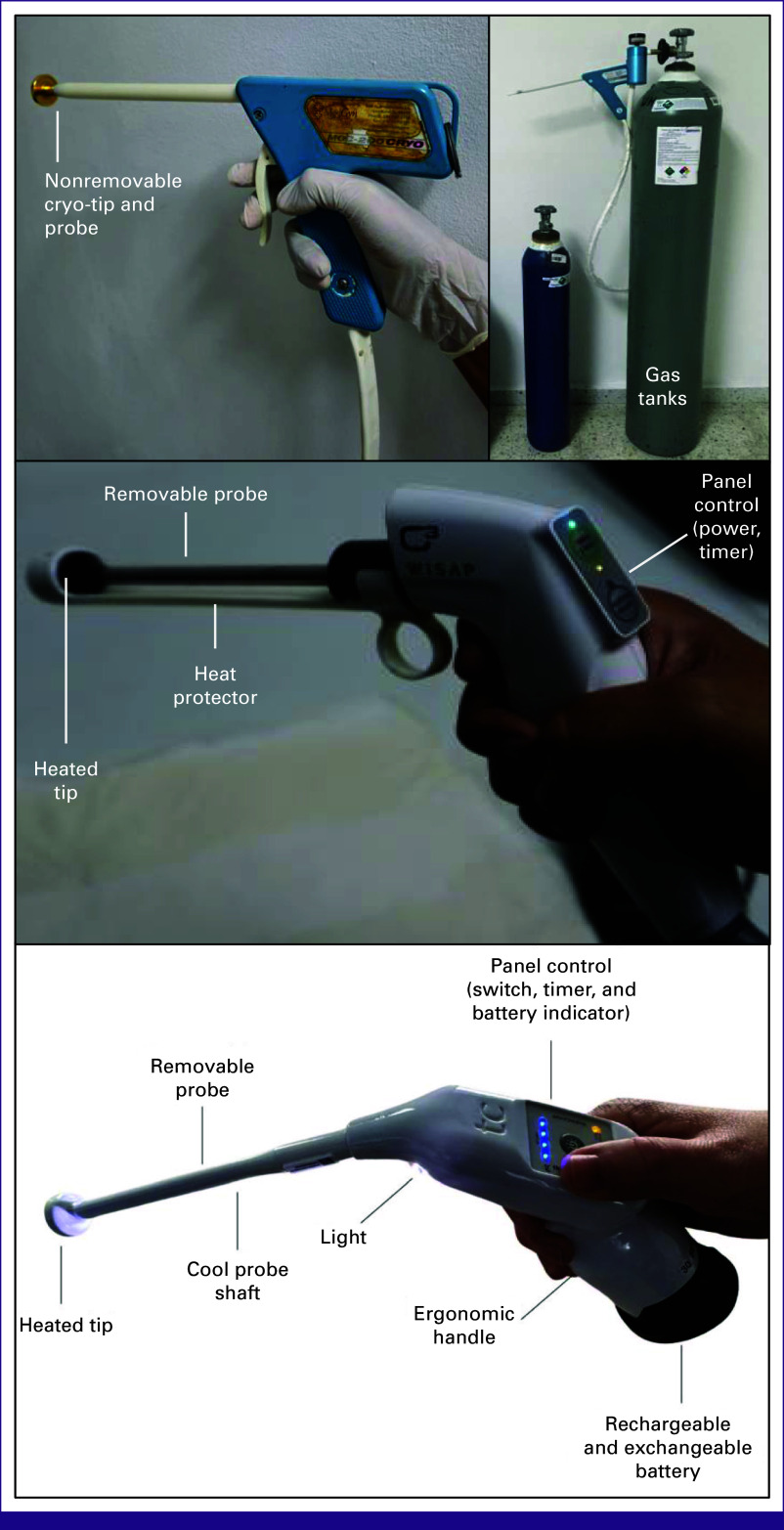
Ablation devices used across studies: gas-based cryotherapy (top panel), WiSAP thermal ablation device (middle panel), and Liger TA device (bottom panel). TA, thermal ablation.

Women across all studies received treatment on the same day of their random assignment by a trained health care provider (ie, a nurse or a clinician). Those assigned to gas-based cryotherapy received the standard double-freeze procedure (3-minute freeze; 5-minute thaw; 3-minute freeze), whereas those assigned to TA received slightly different version of treatment based on the study protocol: (1) single application of a 19-mm conical tip for 40 seconds; (2) application of a 16-mm conical tip for 20 seconds; (3) single application of a 10-mm shallow probe applied to the endocervix for 20 seconds, followed by a 16-mm flat probe applied to the ectocervix for 20 seconds in overlapping fields; and (4) application of a 19-mm or 20-mm flat tip for 30 seconds. All protocols allowed for multiple overlapping applications to cover the entire transformation zone, if needed. None of the study sites applied any local anesthetic to the cervix before TA. A graphic representation of the devices used across these studies is presented in Figure 1.

Across all studies, women were asked immediately after treatment to report their level of pain during the procedure using an analog visual scale immediately after treatment. Health care providers also noted other immediate side effects of the treatment, including vasovagal responses or bleeding. Overall satisfaction with the treatment was assessed during an in-person follow-up interview conducted approximately 2-6 weeks after treatment. Finally, all women were asked to return around 12 months after completing their initial procedure to evaluate treatment efficacy. This assessment included HPV testing and the VIA test in all studies and four-quadrant biopsies in those participants from China, Colombia, and El Salvador.

### Statistical Analysis

The primary outcome used for this analysis was the HPV test result at the 12-month follow-up visit, whereas the primary treatment variable was the main ablation technique used during baseline treatment (ie, gas-based cryotherapy or TA). Secondary outcomes included the level of pain during treatment, the presence of side effects, and satisfaction with treatment. Subgroup analyses were performed using the HIV status and specific TA protocol.

A descriptive analysis for the primary outcome, as well as selected covariates for the secondary outcomes, and subgroup analysis were performed using central tendency measures (mean, median), as well as frequencies and percentages. The differences by primary treatment were tested using the Wilcoxon rank-sum test or Fisher's exact test, as appropriate. Estimates of treatment efficacy based on the follow-up HPV test result are presented with their corresponding 95% CI.

## RESULTS

A total of 2,948 participants were randomly assigned to either gas-based cryotherapy (n = 1,296) or TA (n = 1,652) in their original studies—plus HPV-positive in Zambia—and thus were included in this analysis. The mean age among all participants was 34 years (SD = 9), and the majority had one to two previous pregnancies (56.1%) and one to two vaginal deliveries (51.1%). Among the 1,039 participants who were included from Zambia, 709 (69.4%) were positive for HIV. Additional information is given in Table 2.

**TABLE 2:** Demographic and Clinical Characteristics by Study Site

Variable	China (n = 1,000)	Colombia (n = 62)	El Salvador (n = 847)	Zambia (n = 1,039)
Age, years, mean (SD)	34 (8)	32 (10)	35 (10)	33 (7)
Previous pregnancies, No. (%)				
None	205 (20.5)	10 (16.1)	55 (6.5)	89 (8.6)
1-2	499 (49.9)	34 (54.8)	734 (86.7)	387 (37.2)
3-4	228 (22.8)	12 (19.4)	51 (6.0)	345 (33.2)
5+	68 (6.8)	6 (9.7)	7 (0.8)	218 (21.0)
Vaginal deliveries, No. (%)				
None	254 (31.9)	6 (11.5)	78 (9.8)	115 (11.1)
1-2	472 (59.4)	34 (65.4)	419 (52.9)	444 (42.7)
3-4	66 (8.3)	8 (15.4)	217 (27.4)	331 (31.9)
5+	3 (0.4)	4 (7.7)	78 (9.8)	149 (14.3)
HIV status, No. (%)				
Negative	Unavailable	Unavailable	Unavailable	313 (30.6)
Positive	Unavailable	Unavailable	Unavailable	709 (69.4)

Abbreviation: SD, standard deviation.

Of the 1,296 participants who received gas-based cryotherapy, baseline HPV test results were available for 922 (71%). From those, HPV results were positive in 871 (94.4%), negative in 48 (5.2%), and invalid in three (0.3%). Similarly, test results were available for 986 (60%) participants who received TA. Among them, 929 (94.2%) were HPV-positive, 54 (5.5%) were HPV-negative, and three (0.3%) had invalid HPV results.

Overall, 2,401 (81.4%) participants across all three studies returned to their 12-month follow-up visit (80.9% and 81.8% from those receiving gas-based cryotherapy and TA, respectively). Among those who underwent gas-based cryotherapy and had follow-up HPV test results available, 393 (58.7%) were negative. Similarly, 459 of 734 (62.5%) of those who underwent TA were HPV-negative at follow-up (Table 3). Among participants with a positive test during their 12-month follow-up visit, HPV 16 represented 34.2% of cases, whereas HPV 18/45 was identified in 18.1%. There were no differences in the distribution of HPV genotypes by ablative treatment (Table 3). Finally, as expected, we observed a lower proportion of HPV-negative results during the follow-up visit in those living with HIV compared with their non-HIV counterparts. However, after stratifying by HIV status, there were no substantial differences between TA and gas-based cryotherapy (Table 4). Of note, of 752 (39.4%) participants who tested positive for HPV 16 at baseline, 530 had follow-up results. Among them, 315 (59.4%) were negative, 160 (30.2%) remained positive for HPV 16, and 55 (10.4%) were positive for a new HPV type. Although a larger proportion of remission was found among participants treated with TA compared with gas-based cryotherapy (61.4% *v* 56.3%, respectively), this was not statistically significant using a chi-square test with an alpha level of .05 (*P* value = .2).

**TABLE 3:** HPV Test Results at the 12-Month Follow-Up Visit by Ablation Treatment

HPV Test Result	Overall (n = 2,401)	Gas-Based Cryotherapy (n = 1,049)	Thermal Ablation (n = 1,352)	*P* ^a^
HPV result, No. (%)				.14
Negative	852 (60.7)	393 (58.7)	459 (62.5)	
Positive	547 (39.0)	276 (41.2)	271 (36.9)	
Invalid test	5 (0.4)	1 (0.1)	4 (0.5)	
Test not done	997	379	618	
HPV genotype,^b^ No. (%)				.91
HPV 16	187 (34.2)	92 (33.3)	95 (35.1)	
HPV 18/45	99 (18.1)	51 (18.5)	48 (17.7)	
Other types	261 (47.7)	133 (48.2)	128 (47.2)	

Abbreviation: HPV, human papillomavirus.

^a^ Fisher's exact test.

^b^Among those with a positive HPV test.

**TABLE 4:** Negative HPV Results at the 12-Month Follow-Up Visit by the HIV Status and Ablative Treatment Protocol

Treatment	Overall^a^	HIV status^b^	
		Negative	Positive
Gas-based cryotherapy (all), No. (%)	392/662 (59.2)		
Zambia site only	155/343 (45.2)	60/93 (64.5)	95/250 (38.0)
TA (all), No. (%)	459/727 (63.1)		
TA (single 20-mm)	162/237 (68.4)		
TA (single 16-mm)	80/93 (86.0)		
TA (multiple tips), No. (%)	73/88 (83.0)		
TA (single 19-mm) [Zambia site]	144/309 (46.6)	66/98 (67.3)	78/211 (37.0)

NOTE. 1: Denominators exclude participants with missing data on the HPV test during the 12-month visit, as well as for HIV status in Zambia. 2: TA (single 16-mm) and TA (multiple tips) are part of the same study (ClinicalTrials.gov identifier: NCT03429582).

Abbreviations: HPV, human papillomavirus; TA, thermal ablation.

^a^ Participants from China, Colombia, and El Salvador can be assumed to be HIV-negative based on the overall low prevalence of HIV in those countries and virtually zero prevalence in the centers where recruitment was conducted.

^b^ Only participants from Zambia were tested for HIV status.

In general, participants who underwent TA experienced higher levels of pain compared with those assigned to gas-based cryotherapy. The proportion of women experiencing severe pain (ie, 7-10 in the visual analog scale) with cryotherapy and TA was 3.9% and 7.6%, respectively (Table 5). However, in all cases, pain from treatment was transient and most women had no pain about 5 minutes after treatment. Mild bleeding (spotting) after treatment was also more common in participants receiving TA (25.8% *v* 13.5%), whereas vasovagal responses were more common in those treated with cryotherapy (Table 5).

**TABLE 5:** Side Effects of Treatment and Patient-Centered Outcomes

Side Effect	Overall (n = 2,401)	Cryotherapy (n = 1,049)	Thermal Ablation (n = 1,352)	*P* ^a^
Pain during treatment, No. (%)				<.01
None	714 (29.9)	402 (38.6)	312 (23.2)	
Mild	511 (21.4)	264 (25.3)	247 (18.4)	
Moderate	1,019 (42.7)	335 (32.1)	684 (50.9)	
Severe	143 (6.0)	41 (3.9)	102 (7.6)	
Vasovagal response,^b^ No. (%)				<.01
Yes	180 (7.5)	149 (14.2)	31 (2.3)	
Bleeding, No. (%)				<.01
Yes	429 (20.3)	128 (13.5)	301 (25.8)	
Satisfaction with the procedure, No. (%)				.32
Highly satisfied	2,242 (98.5)	968 (98.1)	1,274 (98.8)	
Neutral	11 (0.5)	5 (0.5)	6 (0.5)	
Less satisfied	24 (1.1)	14 (1.4)	10 (0.8)	
Did not answer	124	62	62	
Recommend to other women, No. (%)				.73
Yes	2,348 (99.7)	1,023 (99.6)	1,325 (99.7)	

^a^ Pearson's chi-squared or Fisher's exact test.

^b^ Included dizziness, flushing, redness, and nausea.

Stratification by the TA protocol showed differences in pain and side effects, particularly with respect to pain during the procedure and bleeding. For instance, participants receiving the multiple-tip protocol had a higher proportion experiencing moderate or severe pain (Appendix Table A1). Similarly, spotting was more common in those assigned to the multiple-tip protocol.

With respect to patient's satisfaction, virtually all women (98.5%) were highly satisfied with treatment and services. Furthermore, an even larger proportion (99.7%) stated that they would recommend their specific treatment to other women. In both the cases, we did not observe differences between gas-based cryotherapy and TA (Table 5).

## DISCUSSION

Over the past decade, TA has emerged as the new standard for treating cervical precancer in LMICs. In this regard, the ACT program served as a valuable forum for exchanging ideas and experiences that contributed to the development or refinement of new devices that are now widely used in low-resource settings.

This study shows the efficacy, safety, and acceptability of TA compared with those of gas-based cryotherapy by assessing various treatment protocols across four countries with differing levels of resources and infrastructure. In China, Colombia, and El Salvador, treatment was performed by trained colposcopists at a second-level hospital that serves rural and urban populations living in the paracentral region of the country. In Zambia, trained nurses provided the treatment at the primary health clinics as part of the ongoing cervical cancer screening program in the country. Notwithstanding differences across settings, the consistency of our findings further reinforces the reliability and effectiveness of TA as a viable treatment alternative. Within this framework, a revision of the 2019 WHO recommendation^7,8^ for the use of TA (a strong recommendation based on low-quality evidence) is warranted to incorporate new evidence, not only from our studies but also from those conducted since the guidelines were developed.

Despite the progress made over the years, questions remain regarding the optimal TA protocol. In our studies, each protocol presented unique advantages that may be better suited to specific circumstances. For example, smaller tips applied for shorter durations resulted in lower levels of pain and spotting, which can be beneficial in settings like El Salvador, where women reported higher median pain levels. However, other factors (eg, provider training level, cultural aspects, etc) may also explain the differences observed beyond the TA protocol itself. Therefore, it is important to interpret these findings with caution. In addition, while differences in HPV negativity at 1-year post-treatment were observed across protocols, these variations could be attributed to the specific conditions of the studies, such as HIV prevalence. However, the possibility of a true difference in treatment efficacy cannot be entirely ruled out. Therefore, it is crucial for groups in countries currently using TA to establish a standardized protocol that can be adopted for training and application. This approach will help ensure that women worldwide receive the most effective treatment possible.

Establishing an optimal protocol for TA—including the size and shape of probes and the duration of each application—will have significant downstream implications. Currently, there is wide variation in terminology of tips. It will be important to standardize the names of the tips to evaluate efficacy. The urgency of reaching this consensus is growing as screen-and-treat practices are likely to expand with advances in rapid HPV tests that provide genotyping information^9^ and machine learning algorithms that enable more precise triage.^10,11^ In light of these developments, there will be a more widespread use of TA because of its simplicity, affordability, and high acceptability.

Another important question concerns the use of TA among women living with HIV (WLWH). Our findings from Zambia indicate that treatment efficacy in WLWH could be substantially lower than that in non-HIV populations, which is consistent with other available evidence.^12,13^ It is hypothesized, however, that treatment efficacy may be influenced by the degree of immunosuppression. Subgroup analyses of CD4 count in studies of gas-based cryotherapy have shown differences in recurrence of CIN2+ lesions.^14^ Future research should seek to validate these findings for TA and identify the most reliable markers for predicting successful treatment in WLWH. This includes exploring the roles of specific HPV genotypes, the microbiome, immunosuppression status, and the interactions among these factors. It is also important to look at trials using biopsy data from WLWH. Treatment efficacy could be influenced by the size and shape of the probes and the use of multiple applications to ensure that the entire transformation zone is treated. Finally, the role of prophylactic ablation in preventing cervical cancer development in this population is yet to be determined. Future studies should explore its feasibility, carefully weighing the potential benefits against concerns about treatment-related side effects.

Our study has several strengths, including the inclusion of three large, randomized trials conducted in four countries with varying levels of resources and infrastructure across the world. Data for assessing both primary and secondary outcomes were collected in a standardized manner, which enabled us to pool the data and draw inferences about the efficacy of TA as a standalone strategy. Nevertheless, the strengths of this study must be considered alongside its limitations. Notably, not all studies included biopsy-confirmed CIN2+ at baseline and follow-up, which limits our ability to infer true cure rates. However, it is more likely that large-scale screening programs will rely on HPV positivity and clearance as indicators for treatment and disease resolution. In addition, HIV status was assessed at only one of our study sites. While it is reasonable to assume that participants at the other sites were predominantly HIV-negative, this limitation prevented us from evaluating the potential impact of varying training levels or TA protocols on the treatment outcomes for WLWH.

Currently, TA represents the most promising strategy for the treatment of cervical precancer in LMICs because of its ease of use, affordability, and portability. As this approach becomes more widely available, particularly when integrated into screen-and-treat programs, it will be crucial to establish the optimal treatment protocol. Future research should prioritize studying TA effectiveness among WLWH to identify those who would benefit the most from this intervention.

## Data Availability

A data sharing statement provided by the authors is available with this article at DOI https://doi.org/10.1200/GO-25-00050.

## APPENDIX

**TABLE A1:** Differences in Pain and Side Effects, by Treatment Arm

Side Effect	Thermal Ablation Protocol			
	ST: 16-mm Conical (20 seconds)	ST: 19-mm Conical (40 seconds)	MT: 10-mm Shallow (20 seconds), Then 16-mm Flat (20 seconds) Overlapping Fields	ST: 19-mm Flat (30 seconds)
Pain during treatment, No. (%)				
None	12 (3.9)	56 (16.2)	19 (6.0)	225 (60.6)
Mild	81 (26.1)	95 (27.5)	71 (22.3)	0 (0.0)
Moderate	182 (58.7)	171 (49.4)	197 (61.9)	134 (36.1)
Severe	35 (11.3)	24 (6.9)	31 (9.7)	12 (3.2)
Vasovagal response,^a^ No. (%)				
Yes	3 (1.0)	24 (6.9)	4 (1.3)	0 (0.0)
Bleeding, No. (%)				
Yes	99 (42.9)	101 (30.2)	100 (44.1)	1 (0.3)
Satisfaction with the procedure, No. (%)				
Highly satisfied	309 (99.4)	275 (96.8)	317 (99.4)	373 (99.2)
Neutral	2 (0.6)	1 (0.4)	2 (0.6)	1 (0.3)
Less satisfied	0 (0.0)	8 (2.8)	0 (0.0)	2 (0.5)
Did not answer	0	62	0	0
Recommend to other women, No. (%)				
Yes	309 (100.0)	328 (99.4)	314 (100.0)	374 (99.5)

Abbreviations: MT, multiple tips; ST, single tip.

^a^ Included dizziness, flushing, redness, and nausea.
